# Advances in nanotechnology for the therapy of bacterial pneumonia

**DOI:** 10.3389/fcimb.2025.1639783

**Published:** 2025-07-28

**Authors:** Zihan Tian, Yuwei Zhang, Jiachen Yun, Weihong Kuang, Jin Li

**Affiliations:** ^1^ Mental Health Center, West China Hospital, Sichuan University, Chengdu, China; ^2^ West China School of Medicine, Sichuan University, Chengdu, China; ^3^ Department of Geriatric, Chengdu Second People’s Hospital, Chengdu, China; ^4^ West China Fourth Hospital, Chengdu, China; ^5^ National Clinical Research Center for Geriatrics, West China Hospital, Sichuan University, Chengdu, Sichuan, China

**Keywords:** nanotechnology, drug delivery systems, organic nanomaterials, inorganic nanomaterial, bacterial pneumonia

## Abstract

Bacterial pneumonia, a life-threatening infection, is the world’s sixth deadliest disease and the top cause of mortality in children under five. Without timely treatment, bacterial pneumonia can escalate to a 30% mortality rate, particularly in high-risk populations. It may also lead to chronic conditions such as pulmonary fibrosis and chronic obstructive pulmonary disease(COPD), along with systemic inflammatory responses that can progress to sepsis and multi-organ failure. Although antibiotics are generally effective against bacterial pneumonia, current treatment approaches remain insufficient due to several barriers, including the lung’s unique mucus barrier, low pH, high oxidative stress, disruption of alveolar surfactants, and accumulation of hypertonic fluid on the airway surface. In addition, following the excessive use of antibiotics, dysbiosis, secondary infections and resistance occur. Nanomaterials can be an effective way to improve therapeutic effects owing to their change on drug size, physicochemical properties, hydrophobicity along with better targeting ability, and controlled localized release. Organic and inorganic substances and their composites are the three main types of nanomaterials to treat bacterial pneumonia. This review presents the latest advancements and constraints of these nanomaterials from a nanotechnology viewpoint with a view to developing therapeutic strategies for bacterial pneumonia.

## 1 Introduction

Pneumonia, the 6th most common cause of death worldwide, is a common and lethal infectious disease. It is also one of the major killer diseases for under-five children (Murray, 2024). It is mostly due to an external pathogen, e.g. bacteria, viruses and fungi (Ferrer et al., 2018). Bacterial pneumonia, a common lung infection caused by various bacteria (Fu et al., 2024; Safdarpour et al., 2022), is a severe disease with high-severity, high-complication and high-annual-mortality, raising a global concern (Dietert et al., 2017). The primary causative agents are *Streptococcus pneumoniae, haemophilus influenzae, staphylococcus aureus* etc (Willyard, 2017). When the body’s defenses are weakened, pathogenic bacteria usually transmit through the respiratory system, penetrate the lung barrier and cause infections (Yu et al., 2023; Liu et al., 2018; Stockley, 1998). Though prevalence varies by area and population, bacterial pneumonia is more common in children, the elderly, people with weakened immune systems, and people with chronic illnesses (Liu et al., 2023).

Many factors, such as the patient’s age, health status, the type of causative agent, and the promptness and efficacy of treatment, affect the prognosis of bacterial pneumonia (Pletz et al., 2012). Without timely and efficient treatment, it can result in life-threatening complications or even death. It is estimated that bacterial pneumonia can be up to 30 percent lethal if left untreated (Li et al., 2016; Hug and Rossi, 2001).

The primary cause of bacterial pneumonia is the pathogenic bacteria’s destruction of lung tissue, which results in respiratory dysfunction. Furthermore, bacterial pneumonia can induce a systemic inflammatory response that leads to severe outcomes such as sepsis and multi-organ failure (Bergogne-Bérézin and Towner, 1996). Pneumonia may also be the cause of long-term lung conditions like pulmonary fibrosis and chronic obstructive pulmonary disease (COPD) (Guzek et al., 2017; Liu et al., 2022, 2018).

A favorable prognosis for bacterial pneumonia depends on timely and efficient treatment because of its extremely high incidence and potentially dangerous outcomes (Grief and Loza, 2018). Antibiotic therapy is the mainstay of the current treatment. In addition to being limited by traditional drug application dilemmas such as bacterial resistance development and antibiotic-derived dysbiosis and secondary infections, the complex microenvironment of the lungs also affects drug efficacy. Traditional drug delivery is ineffective for the following reasons: the respiratory tract’s mucus layer acts as a dynamic barrier; lipoprotein complexes of active substances on the alveolar surface adsorb hydrophobic drug molecules; the localized acidic environment (pH=5.5-6.5) prevents transmembrane transport of weakly alkaline drugs; and hypertonic fluid formation on the airway surface decreases the solubility of water-soluble drugs and changes their conformation. This gives the conventional drug delivery method the opportunity to be improved in order to fully utilize anti-inflammatory, anti-infective, and organism protection properties (Chen et al., 2021).

The challenges mentioned above can be addressed through nanotechnology. By modifying drug size, surface properties, and hydrophobicity, nanotechnology enables more efficient drug delivery, allowing for targeted transport and precise controlled release. This can significantly improve therapeutic efficacy, reduce the risk of drug resistance, and limit nonspecific inflammatory damage (Tang et al., 2022). Thus, one of the most crucial instruments for treating bacterial pneumonia in the future is the investigation of safer and more effective nanomaterials as well as the best clinical practices for them.

**Figure 1:**
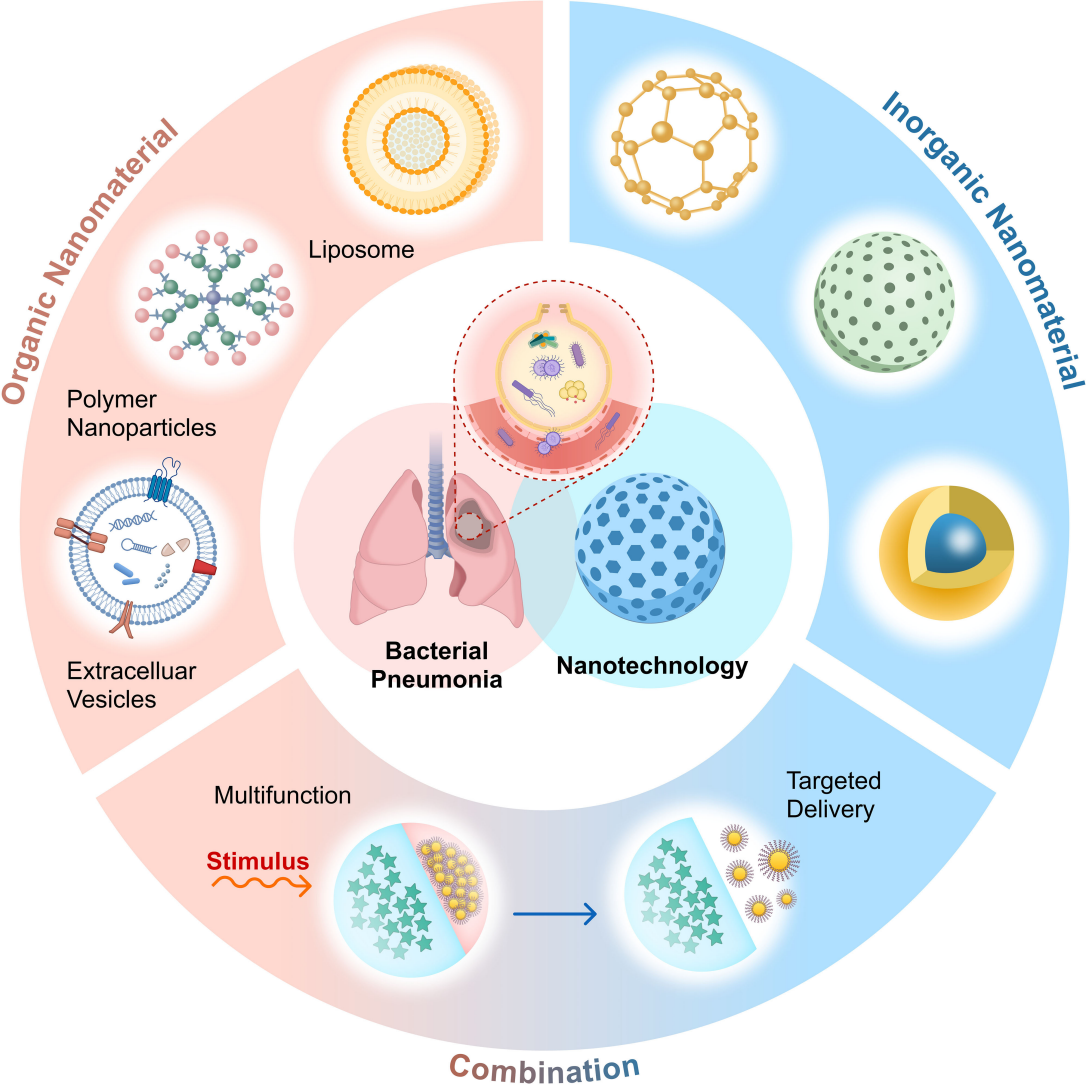
Applicable nanomaterials for the treatment of bacterial pneumonia include organic nanomaterials (such as liposomes, polymers, extracellular vesicles, etc.), inorganic nanomaterials, and their combinations. These nanomaterials achieve targeted drug delivery through distinct mechanisms, thereby improving existing treatment strategies. Created with Figma.

Organic, inorganic, and organic-inorganic composite nanomaterials are the three types of nanomaterials currently used to treat bacterial pneumonia (**Figure 1**). Organic nanomaterials mainly include liposomes, polymeric nanomaterials and extracellular vesicles(EVs). Liposomes can improve the effectiveness of drug therapy by surface modification to achieve precise delivery ensuring sufficient drug delivery to the corresponding lesion; polymer nanomaterials have the advantages of surface modification to achieve multifunctionality, integrating multiple response mechanisms, enhancing drug stability, etc., and some of the materials also have direct antimicrobial activity. In order to improve the immune response specific to antimicrobials and decrease non-specific inflammatory damage, EVs have the ability to regulate the degree of inflammation. The inorganic nanomaterials rely on surface modification, structural design, metal ion selection, and photodynamic characteristics to put together various mechanisms of antimicrobial activity. Additionally, there are promising application prospects for organic-inorganic composite nanomaterials, which can be modified to achieve the superposition of advantages and complementary disadvantages. In addition, medical devices or drugs that can be used for clinical treatment need to be non-toxic (or low toxicity) and good biocompatibility, and these requirements make nanomaterials irreplaceable advantages in the treatment of bacterial pneumonia.

For a comparison between the major categories of nanomaterials mentioned in this paper, please refer to **Table 1**.

**Table 1:** Summary of Types, Advantages and Limitations of Nanomaterials Applied in the Treatment of Bacterial Pneumonia.

Material types	Key advantages	Major limitation
Liposomes	- Targeted delivery - Long-acting release - Low toxicity - Biocompatibility - Degradability - High drug encapsulation capacity - Environment-responsive-release	- Nebulization may alter the physical properties of liposomes and their intracellular deposition, resulting in insufficient effective drug concentrations - Phagocytized by macrophages - Limited penetrable barriers - Insufficient safety-related data - Complex preparation process - Challenging for clinical translation
Polymeric nanomaterials	- Physicochemical properties can be customized - Stimuli-responsive - The degree of polymerization alters permeability and regulates surface charge	- Nanoparticles tend to aggregate due to high surface energy, which affects the stability of materials - Difficult to regulate properties - Limited penetrable barriers - Insufficient safety-related data - Complex preparation process and high cost - Unknown environmental impact
Extracelluar vesicles	- Metabolizable - Biocompatibility and low immunogenicity - Low toxicity - Barrier penetration ability - Specific binding to target cells - Regulate inflammation, promote regeneration, tissue repair, etc. - Stabilizing and protective effects of the lipid bilayer - Potential for engineering modification	- Different components from different sources, with a lack of unified quality control standards - Limitations in *in vivo* clearance and distribution&unmodified ones are prone to clearance - Lack of precise stimulus-responsive release mechanisms - Some mechanisms of action remain unknown - Difficulties in preparation and purification - Challenges in clinical translation
Inorganic nanomaterials	- High biological selectivity - Multifunctional integration - Environmental stability - Excellent photothermal/photodynamic performance	- The chiral generation mechanisms of some materials remain poorly understood - Insufficient penetration depth of near-infrared light in tissues - Prone to be cleared by the endothelial system - Unclear metabolic pathways of some materials - Insufficient safety-related data - Difficulties in preparation and standardization - Challenges in clinical translation
Organic- inorganic composite nanomaterials	- Multimodal synergistic antibacterial activity - Complementary properties of organic and inorganic materials - High designability	- Risks of toxicity and accumulation - Susceptible to degradation - Challenges in clinical translation

## 2 Organic nanomaterials

### 2.1 Liposome

Liposomes are amphiphilic nanovesicles with a hydrophilic core and a hydrophobic lipid layer. The aqueous core is encapsulated by a phospholipid bilayer, which is made up of a bilayer membrane of phospholipids and cholesterol. These particles can encapsulate hydrophilic, hydrophobic, and amphiphilic drugs simultaneously; their particle sizes typically range from 50 to 1000 nm, and nanoscale liposomes (<150 nm) can improve tissue penetration and prolong *in vivo* circulation time. Liposomes can prolong the circulating half-life of drug and achieve microenvironment-specific release by increasing drug stability. As a legally approved nanomaterial, liposomes have some utility and promise for advancement in the management of bacterial pneumonia.

Antibiotics, anti-inflammatory medications, and nucleic acids are just a few of the many biologically active substances that liposomes can encapsulate and transport (Haddadzadegan et al., 2022; Panthi et al., 2024; Caddeo et al., 2013; Meng et al., 2024). Its advantages as a delivery vehicle include the following:

Firstly, liposomes, which are particularly suitable for hydrophobic drugs, can significantly increase drug encapsulation rates through physical encapsulation, chemical gradient methods, and other techniques. By improving the solubility and dispersion of poorly soluble compounds, liposomes allow drugs that are otherwise difficult for the body to absorb and utilize to become more bioavailable (Muralidharan et al., 2015). Their structure also contributes to increased drug stability, protects the encapsulated compounds from the *in vivo* environment, and prevents degradation or inactivation of the drug (Kumari et al., 2023). For example, andrographolide (AG), despite having multiple therapeutic properties, including antiviral, antibacterial, and anti-inflammatory effects, is insoluble in water. When used with a dry powder inhaler for pulmonary drug delivery, AG encapsulated in liposomes forms a phospholipid vesicle structure that significantly improves its solubility. This enables the drug to reach the deeper regions of the lungs, achieving a large absorption area and high membrane permeability of lung tissue (Ju et al., 2017; Muralidharan et al., 2015; Simon et al., 2016; Li et al., 2017).

**Figure 2:**
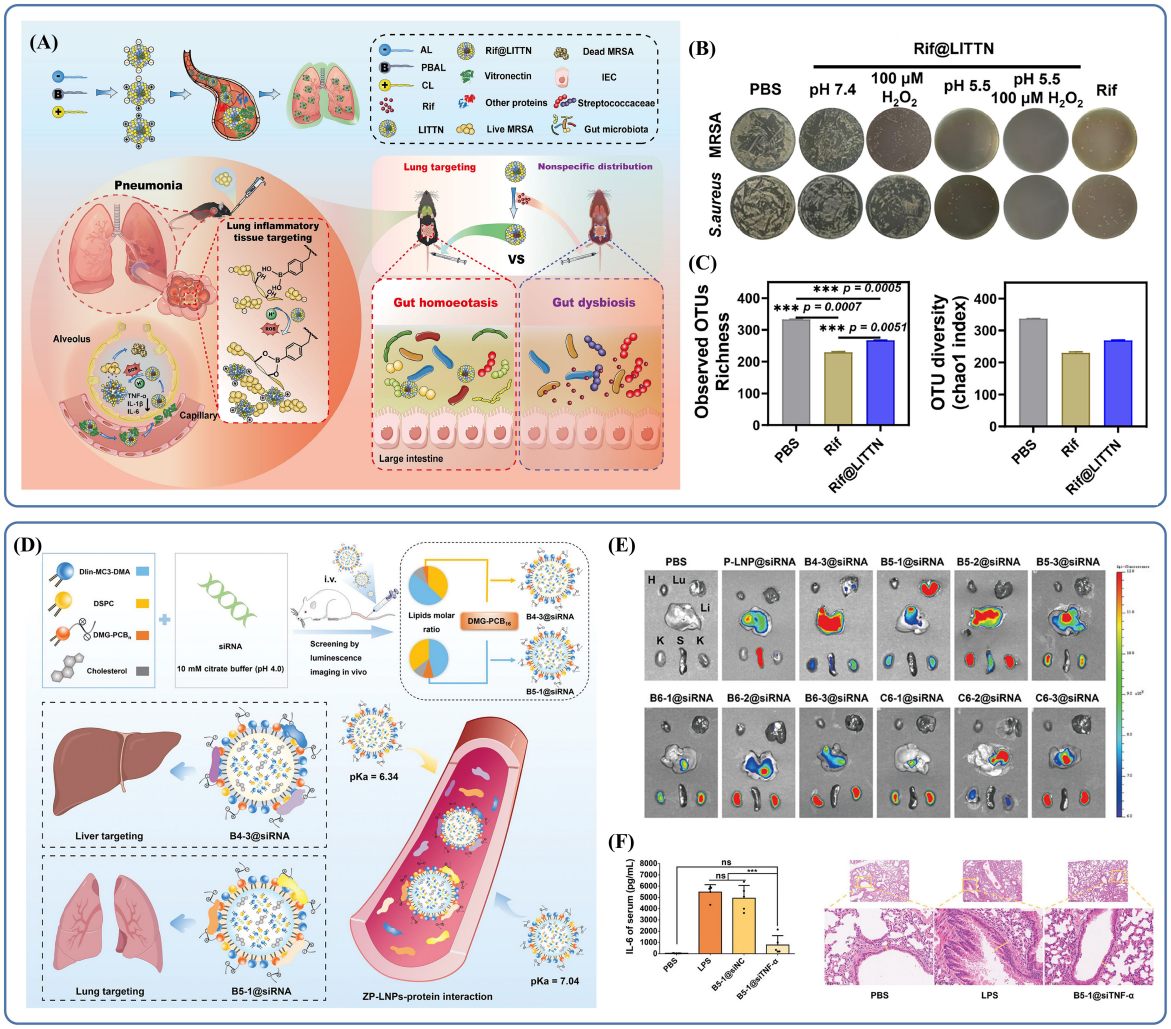
**(A)** A modified liposomal nanoparticle, LITTN, effectively treats bacterial pneumonia while preserving gut bacterial diversity. Reproduced by permission. Copy right 2024, Elsevier. **(B)** Under hyperoxic and acidic conditions, Rif@LITTN leads to a reduction in the number of bacterial colonies of *Staphylococcus aureus* and *MRSA* strains on agar plates. Reproduced by permission. Copy right 2024, Elsevier. **(C)** The α-diversity of bacteria treated with Rif@LITTN was higher than that in the free Rif group. Reproduced by permission. Copy right 2024, Elsevier. **(D)** Schematic illustration of the construction and delivery of ZP-LNPs. Reproduced by permission. Copy right 2024, Wiley. **(E)** *In vivo* luminescence imaging revealed that the two ZP-LNPs were primarily distributed in the liver and lung. Reproduced by permission. Copy right 2024, Wiley. **(F)** Treatment with B5-1@siTNF-α reduced inflammation, and the alveolar structures remained relatively more intact. "***" signifies an extremely statistically significant difference (P < 0.001). Reproduced by permission. Copy right 2024, Wiley.

Second, to reach targeted delivery, increase therapy efficacy, and lower side effects, liposomes can be surface-modified with folic acid (FA), antibodies, or peptides. For example, lung inflammatory tissue-targeted nanoparticles (LITTN) are engineered with cationic and phenylboronic acid-modified lipids on their surface (Fu et al., 2024). By chemical modifications, structural change achieve targeted delivery: coordination between boric acid and cis-diol groups allows for bacterial recognition and improved targeting, the positively charged cationic lipids interact with negatively charged bacterial membranes, improving drug attachment at the infection site (Monopoli et al., 2011) (Zhang et al., 2018). LITTN has demonstrated good lung-targeting ability *in vivo*; following intravenous injection, it accumulates in the lungs while decreasing distribution to other organs (Dilliard et al., 2021). Misuse of antibiotics is concerning, as it can lead to disruptions in the intestinal flora, which has systemic implications. Delivering nanoparticles(NPs) that specifically target the lungs helps protect gut microbiota from antibiotic-induced shifts in abundance, diversity, and community structure (**Figure 2A**), indicating a favorable safety profile (Thänert et al., 2022; Zhang et al., 2022b). In animal studies, mice treated with rifampicin-loaded LITTN showed greater gut microbiota diversity compared to those given free rifampicin. Rif@LITTN also exhibited stronger antimicrobial activity than free rifampicin in *in vitro* assays under inflammatory conditions characterized by low pH and oxidative stress (**Figure 2B**), suggesting improved targeting and bactericidal performance. Microbiota analysis of fecal samples revealed that the Rif@LITTN-treated group maintained higher α-diversity than the group receiving free rifampicin (**Figure 2C**).

It is worth noting that appropriate surface modifications can alter a drug’s organ-targeting profile, enabling more precise delivery and allowing repurposing of existing materials, which helps reduce costs. For example, small interfering RNAs (siRNAs) hold significant potential for treating bacterial pneumonia, especially in controlling inflammation, as they can suppress target mRNA expression and thereby reduce corresponding protein levels. Lipid nanoparticles (LNPs), which are clinically developed siRNA carriers, facilitate siRNA delivery into the cytoplasm while protecting it from degradation during transport. Although LNPs are primarily used in clinical applications to target the liver, they can be further modified to improve targeting of extrahepatic tissues. LNPs are typically composed of four key elements: ionizable cationic lipids, polyethylene glycol (PEG) lipids, cholesterol, and accessory lipids. Ionizable cationic lipids are responsible for siRNA adsorption under acidic conditions and promote endocytosis or lysosomal escape of siRNA by interacting with endosomal and lysosomal membranes after the LNP is taken up by the target cells (Han et al., 2021a; Patel et al., 2019). Co-lipids contribute to overall stability by self-assembling into lipid bilayers with high phase transition temperatures (Trollmann and Böckmann, 2022). Cholesterol improves the structural integrity of the LNP (Jia et al., 2024). PEG lipids play a role in preventing aggregation and undesirable interactions with the biological environment, allowing LNPs to evade phagocytic clearance and prolong their *in vivo* circulation time. As an example, Zwitterionic polymer LNPs (ZP-LNPs) show how these material design principles can be applied to develop more effective delivery systems.

ZP-LNPs are constructed using the zwitterionic polymer polycarboxybetaine (PCB)-modified 1,2-dimyristoylglycerol (DMG) lipid, referred to as DMG-PCB_n_. These structures enable selective delivery of siRNAs to both the liver and lungs (**Figure 2D**) (Li et al., 2025). By adjusting parameters such as the lipid-to-siRNA mass ratio, the molar ratio of lipid components, and the degree of polymerization of DMG-PCB_n_, researchers developed libraries of ZP-LNPs, allowing for screening and selection based on specific therapeutic goals. Mechanistic studies suggest that organ-specific delivery patterns and the *in vivo* fate of ZP-LNPs are largely governed by pKa values and the composition of the protein corona. *In vivo* imaging demonstrated that the B5-1@Cy5-siRNA subtype accumulates specifically in the lungs (**Figure 2E**), showing that different modifications lead to clear differences in organ distribution and enable selective targeting. ZP-LNPs primarily reduce inflammation by suppressing target genes and decreasing the levels of inflammatory mediators like TNF-α. Pathological analysis showed that B5-1@siTNF-α treatment in mice with lung inflammation preserved alveolar architecture, reduced bronchial wall thickness, and significantly decreased infiltration of inflammatory cells (**Figure 2F**). Moreover, the serum level of IL-6 in lipopolysaccharide(LPS)-stimulated mice was significantly elevated compared to normal controls, further supporting the accuracy and effectiveness of this delivery strategy.

Thirdly, liposomes exhibit strong biocompatibility. Their phospholipid bilayer structure resembles that of cell membranes, making them non-toxic, non-immunogenic, and suitable for multiple administration routes, including intravenous injection and inhalation (Li et al., 2017; Yu et al., 2023; Pal et al., 2017). Inhalation of andrographolide dry powder inhalers (LADPIs) diminishes inflammatory injury. LADPIs reduce the levels of inflammatory biomarkers such as IL-1, TGF-β, total proteins and lower the recruitment of leukocytes and neutrophils (Li et al., 2017). In addition to drug delivery, liposomes also reduce damage to extrapulmonary organs and prevent unwanted drug accumulation outside the lungs (Rommasi and Esfandiari, 2021).

To examine the safety of liposome-based treatment for bacterial pneumonia, the health condition of experimental animals was observed in the liposome-treated group. Tissues from organs stained with H&E did not show structural or morphological abnormality. No significant difference was observed in basic physiological parameters including mental status, mobility and weight of treated and control groups. According to the findings, administration of liposomes did not interfere with the normal structure and function of any other major organ (Meng et al., 2024).

### 2.2 Polymeric nanomaterials

Most polymer NPs are made of polymer compounds with repeating units. Polymer nanotechnology is making a mark in the treatment of bacterial pneumonia by offering better control over drug release. Researchers accomplished this by adjusting parameters like polymer molecular weight, adding functional modifications, and degrading esters and similar links. These actions result in small molecules that are not metabolically toxic. The delivery of various drug forms, such as hydrogels, microspheres, NPs containing small molecules, proteins, and nucleic acids are also possible with polymer-based systems. These characteristics provide answers to problems like drug resistance and inefficiencies in standard antibiotic treatment (Lim et al., 2016). Scientists have made many types of nanocarriers in the form of a polymer to enhance the activities of existing drugs. These systems enhance the efficiency of delivery, help cross the physical barrier of the lung, and exhibit greater antimicrobial activity compared to the drug that has not been modified (Solanki et al., 2024). In some instances, the polymeric materials formulate complexes with the drugs to help deliver them and lessen their toxicity systemically (Verma et al., 2024).

**Figure 3:**
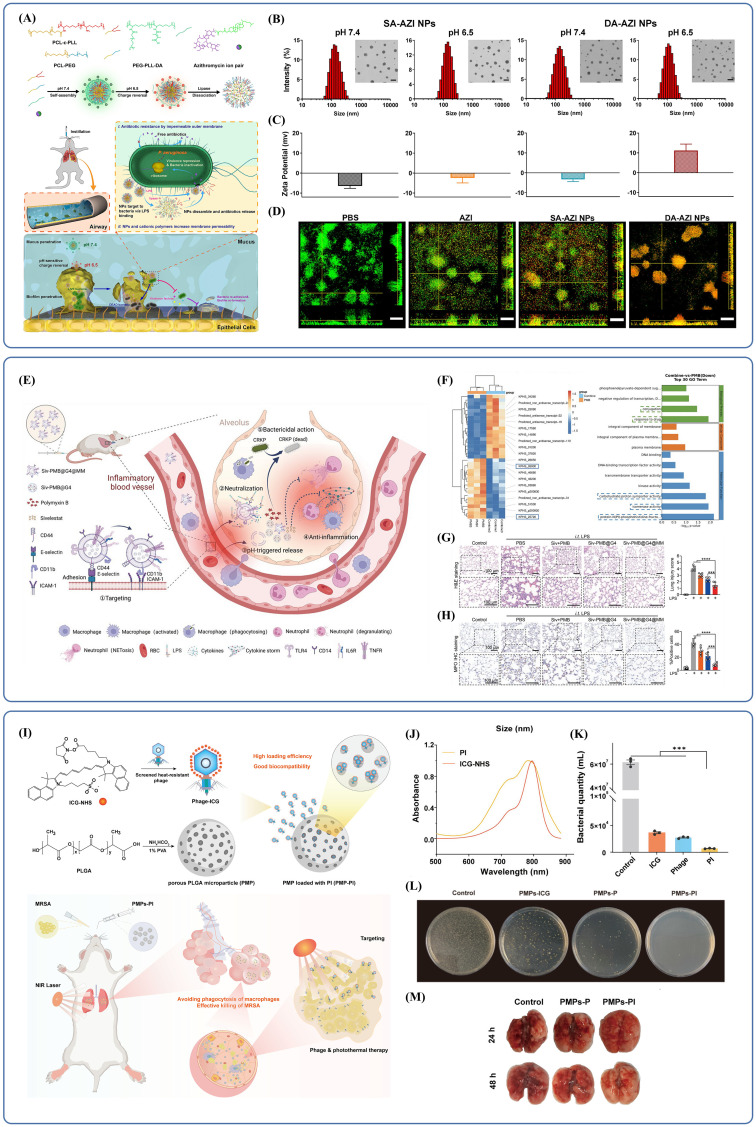
**(A)** Schematic illustration of the preparation and pH/Lipase-responsive behaviors of DA-AZI NPs, and their application to pulmonary infection management. Reproduced by permission. Copy right 2024, Elsevier. **(B)** DA-AZI NPs were uniformly dispersed and had a spherical morphology. Reproduced by permission. Copy right 2024, Elsevier. **(C)** In pH=7.4 PBS, the NPs displayed a slightly negatively charged surface. DA-AZI NPs exhibit charge reversal, while SA-AZI NPs maintain a negative charge regardless of pH. Reproduced by permission. Copy right 2024, Elsevier. **(D)** Effectiveness of two NPs in disrupting biofilms. Reproduced by permission. Copy right 2024, Elsevier. **(E)** Schematic illustration of the lung targeting and anti-infection effect of Siv-PMB@G4@MM against severe CRKP pneumonia. Reproduced by permission. Copy right 2024, Wiley. **(F)** The upregulated and downregulated genes after combination therapy. Reproduced by permission. Copy right 2024, Wiley. **(G)** The Siv-PMB@G4@MM group exhibited the least inflammatory changes and the most intact alveolar structure. Reproduced by permission. Copy right 2024, Wiley. **(H)** Siv-PMB@G4@MM can effectively alleviate the high expression of MPO in lung tissue. Reproduced by permission. Copy right 2024, Wiley. **(I)** Schematic illustration of the construction of PLGA microspheres loaded with ICG-Conjugated Phage and its use in the treatment of *MRSA* Pneumonia. Reproduced by permission. Copy right 2024, American Chemical Society. **(J)** The spectrum of PI was significantly broadened. Reproduced by permission. Copy right 2024, American Chemical Society. **(K)** The PI group demonstrated stronger *in vitro* bactericidal efficacy against bacteria including *MRSA*. Reproduced by permission. Copy right 2024, American Chemical Society. **(L)** Only under light irradiation did the PMPs-ICG group exhibit bactericidal efficacy. Reproduced by permission. Copy right 2024, American Chemical Society. **(M)** Compared with other groups, the PMPs-PI group showed a better therapeutic effect. "***" signifies an extremely statistically significant difference (P < 0.001). Reproduced by permission. Copy right 2024, American Chemical Society.

The physicochemical properties of polymer NPs are unique and make them ideal tools for pulmonary drug delivery. Through suitable modifications in surface properties these NPs can also release an active ingredient in a controlled manner at the target site. Cationic NPs can enhance drug uptake by interfering with negatively charged bacterial or cellular membranes in particular. Li et al. developed a microenvironment-responsive, charge-switchable NPs system (DA-AZI NPs) (Li et al., 2024b). This was achieved by first synthesizing an amphiphilic polymer, polycaprolactone-epsilon-poly(L-lysine) (PCL-ϵ-PLL), which was co-assembled with polycaprolactone-poly(ethylene glycol) (PCL-PEG) into NPs. A pH-sensitive, negatively charged polymer, dimethylmaleic anhydride (DA)-modified poly(ethylene glycol)-epsilon-poly(L-lysine) (PEG-PLL-DA), was synthesized using cholesteryl hemisuccinate (CHEMS) as a counterion and used to coat the positively charged NPs, effectively masking their surface charge. The resulting DA-AZI NPs demonstrated the ability to rapidly pass through airway mucus and accumulate at infection sites within biofilms (**Figure 3A**). In acidic microenvironments, such as those found in biofilms, the amide bond between ϵ-PLL and the amino group of DA was cleaved, causing a charge reversal. This allowed the positively charged core to be exposed, while SA-AZI NPs, which remained negatively charged regardless of pH, detached from the NPs surface due to electrostatic repulsion (**Figure 3B, C**). The exposed ϵ-PLL-derived NPs then penetrated deep into the biofilm, targeted *P. aeruginosa* through LPS binding, and increased bacterial membrane permeability. Within 20 minutes, DA-AZI NPs were broadly distributed throughout the biofilm, and by 40 minutes, they had penetrated nearly the entire structure (**Figure 3D**). The azithromycin was then precisely released at the infection site, successfully overcoming resistance in highly drug-resistant *P. aeruginosa*.

In recent years, bionic nanoplatforms have gained increasing attention due to their ability to effectively target inflammatory sites while avoiding immune surveillance. One such biomimetic nanocarrier, Siv-polymyxin B (PMB)@G4@MM, is constructed by encapsulating a polymeric core with macrophage membranes (MMs) (Duan et al., 2024). These membranes not only direct the nanocarrier to inflammation sites but also help capture LPS and pro-inflammatory cytokines (Gao et al., 2020; Tan et al., 2021; Yin et al., 2023; Cao et al., 2022). The polymeric core is composed of generation 4.0 polyamidoamine dendrimer (G4 PAMAM or G4), which can accommodate both lipid-soluble drugs (Siv) and water-soluble drugs (PMB) within its internal cavities. These drugs are released in response to low pH in acidic microenvironments and are taken up by endocytosis. Additionally, G4 carries intrinsic antimicrobial activity due to its strongly positive surface charge, supporting its potential use against *CRKP* (Han et al., 2023; Chen et al., 2023; Li et al., 2021) (**Figure 3E**). Subsequent studies have confirmed the synergistic antibacterial effect of G4 combined with PMB. Transcriptome sequencing (RNA-seq) revealed significant shifts in gene expression profiles. In the co-treatment group, genes associated with drug response were significantly downregulated, hindering the transfer of bacterial resistance genes. There was also reduced expression of genes linked to isoenzyme activity, which affects bacterial adaptability under extreme conditions, as well as genes involved in energy metabolism. These changes accelerated bacterial collapse, contributing to the observed synergistic antibacterial effects (**Figure 3F**). In *in vivo* experiments, the Siv-PMB@G4@MM group showed minimal lung injury and a reduced inflammatory response (**Figure 3G**). Quantitative analysis and immunohistochemical staining for myeloperoxidase (MPO), a marker of neutrophil infiltration, revealed significantly lower MPO expression in lung tissues from this group (**Figure 3H**), indicating superior antibacterial and anti-inflammatory effects. Another approach involves active delivery. The biohybrid microrobot, known as the algal-NP-robot, is made by attaching drug-loaded polymer NPs encapsulated in neutrophil membranes to Chlamydomonas reinhardtii (Zhang et al., 2022a). Its biomimetic design helps reduce immune clearance. Powered by the propulsion of living microalgae cells, this system generates strong and sustained motion, enabling a distinctive form of targeted drug delivery.

Another strategy is to target ligand modification. By attaching specific ligands to the surface of polymer NPs, drug accumulation at the disease site can be increased, concentration at the target area improved, and systemic distribution reduced. Subsequently, this helps limit toxicity to other organs (Elnakat and Ratnam, 2004). For example, as inflamed lung tissue tends to overexpress folate receptors (FRs), FA-modified NPs have been applied for targeted antibiotic delivery to macrophages (Puligujja et al., 2013), allowing for more accumulation of the drug in affected regions. Due to their chemical properties, polymeric NPs are also able to cross the lung’s mucus barrier, reach deep into the infection site, and bind the drug to achieve local concentration at the lesion. One example is MXF/Oxi-αCD NPs, formed by encapsulating moxifloxacin with nanomaterials capable of penetrating mucus through PEG coating (Wang et al., 2019). These polymeric NPs release the drug rapidly in infected microenvironments, facilitated by their design, which includes responsiveness to reactive oxygen species (ROS). In a different example, a nanotherapeutic modified by a heteropolyvalent ligand goes through biofilms and releases its drug inhibiting drug-tolerant strains resisting to stresses. Results indicated that the system can disperse more than 80% biofilm when exposed to laser light (Zhao et al., 2019). In addition, the azithromycin (AZM) incorporation into N-fumaroylated diketopiperazine (FDKP) microparticles improved the aerodynamic performance of AZM@FDKP-MPs. As a result, the drug has improved chances of reaching the deeper regions of the lung (Wang et al., 2018).

Polymeric nanomaterials can also deliver biotherapeutics. One of the most promising strategies for dealing with bacterial infections is phage therapy which has clinical translational potential, safety and efficacy. Yet, its utilization was limited by poor retention and delivery policy barrier. To solve this problem, Liu et al. designed poly(lactic-co-glycolic acid) (PLGA) microspheres for dry powder inhalation in a synergistic antimicrobial approach (Wang et al., 2024a; Kumbhar et al., 2022; Xu et al., 2024; Yu et al., 2021). The porous structure of these microspheres was loaded with phages modified on the surface by indocyanine green (ICG), forming a composite referred to as PMP-PI. ICG is a *in vivo* applicable dye that penetrates tissue and generates heat under radiation, enabling a combination of phage and photothermal therapy for bacterial pneumonia (Liu et al., 2024)(**Figure 3I**). Notably, although the NIR absorption peaks of the coupled phage-ICG complexes (PIs) and free ICG were similar, the modified PIs had a broader absorption spectrum, which improved photothermal conversion efficiency (**Figure 3J**). In the absence of laser irradiation, no sterilization effect was observed, confirming that the photothermal response was the primary antimicrobial mechanism. In *in vitro* experiments under laser irradiation, the PI group showed stronger bactericidal activity against *MRSA* compared to other groups (**Figure 3K**), and the PMP-PI also demonstrated robust sterilization when irradiated (**Figure 3L**). In *in vivo* tests, the PMP-PI group showed better therapeutic outcomes than the PMP-P group, as indicated by healthier lung tissue appearance, reduced hemorrhage, lower lung weight, and less edema (**Figure 3M**). These findings suggest that this delivery method not only achieves effective and precise antibacterial action but also maintains safety under *in vivo* conditions.

Polymeric nanomaterials offer advantages that go beyond drug delivery alone. They present new approaches to the ongoing challenge of drug selection caused by bacterial resistance. These materials not only improve the antibacterial effects of loaded drugs but can also exhibit direct antibacterial activity themselves (Zhou et al., 2019). Guanidine-functionalized polycarbonate is a broad-spectrum, biodegradable antimicrobial agent. Due to its unique mechanism of cytoplasmic precipitation, which occurs after membrane translocation, it is able to kill bacteria effectively and does not promote resistance development even after repeated use (Chin et al., 2018). Increasing the length of the hydrophobic alkyl spacer in the polymer backbone significantly increases the hydrophobicity and hence more micelles formation. The hydrophobic domains of the polymer are protected by these micelles inside the core, which enhances antibiotic activity and decreases toxicity and non-specific interactions with plasma components after intravenous administration (Yang et al., 2019).

Polymeric NPs also help in stabilizing therapeutic agents and apart from prolonging their half-life, also protect them from internal and external degradation (Janes et al., 2001). Chitosan NPs can effectively deliver Cpl-1, a phage-derived endolysin with low bioavailability in the body. These biocompatible, low immunogenic, non-toxic, and adhesive carriers can efficiently shield Cpl-1, improving its bioavailability (Gondil et al., 2020). Using electrostatic interactions, a unique polyionic nanocomplex was made from positively charged PMB and negatively charged DA-grafted CS. The PMB is protected from electrostatic binding, which retains its antibacterial effect, and dampens its neurotoxic and nephrotoxic effects (Chai et al., 2020). For detailed structural characteristics, drug loading, delivery mechanisms, sensitive bacteria, size and zeta potential of these polymeric NPs, please refer to **Table 2**.

**Table 2:** Polymeric nanomaterials for the treatment of bacterial pneumonia: their structural compositions, drug loading, and delivery mechanisms.

Material	Drug	Delivery Mechanism	Sensitive Bacteria	Reference	Size	Zeta Potential(mv)
PCL-ϵ-PLL+PCL- PEG+CHEMS-AZI	AZI	pH-responsive charge-switching; inner-layer cationic particles binding to bacterial biofilms	*P.aeruginosa*	(Li et al., 2024b)	131 ± 4.2 nm	-3.29 ± 1.11
G4 PAMAM+MM	Siv & PMB	Receptor- and charge-mediated targeting by MM; acidic microenvironment-triggered release; avoidance of drug degradation by lysosomes	*CRKP*	(Duan et al., 2024)	140nm	-12.60 ± 1.95
Chlamydomonas reinhardtii microalgae+PLGA + neutrophil membrane	Cip	Active delivery	*P.aeruginosa*	(Zhang et al., 2022a)	105.8-110.3 nm	-22.5--19.5
HPAP-modified cyclodextrin	MXF	Sputum penetration; FR-mediated macrophage targeting; Controlled payload release in high-ROS microenvironment	*P.aeruginosa*	(Wang et al., 2019)	254.2 ± 9.5 nm	− 32.4 ± 0.4
Shell:heteromultivalent glycomimetics Core: acid-sensitive polymers	Phototherapeutic agent	pH-tunable ON/OFF NPs; Targeting bacterial lectins;Acid-responsive release	*P.aeruginosa*	(Zhao et al., 2019)	171–187 nm	9.5-12.2
FDKP	AZM	Dry powder inhalation	*S. pneumoniae*	(Wang et al., 2018)	D90=8.255 ± 0.458 nm	–
PLGA	ICG-NHS-modified phage+PTT	Dry powder inhalation. Irradiation via laser penetration through tissue	*MRSA*	(Liu et al., 2024)	10.67 μm	-31.7
Guanidinium-functionalized polycarbonates	_	Intravenous injection	*K. pneumoniae*	(Yang et al., 2019)	–	–
Chitosan	Cpl-1	Prolong the half-life and improve bioavailability	*S. pneumoniae*	(Gondil et al., 2020)	127 ± 28 nm	+36.9
DA&CS	PMB	pH-responsive and electric charge-switched release	*P.aeruginosa*	(Chai et al., 2020)	D_h_= 154.2 ± 1.4 nm	-12.8 ± 0.9

PCL, polycaprolactone; PLL, poly(l-lysine); PEG,poly (ethylene glycol); CHEMS, cholesteryl hemisuccinate; AZM, azithromycin; P.aeruginosa, Pseudomonas aeruginosa; G4 PAMAM, generation 4.0 polyamidoamine dendrimer; MM, macrophage membrane; Siv, Sivelestat; PMB, Polymyxin B; CRKP, carbapenem-resistant Klebsiella pneumoniae; PLGA, poly(lactic-co-glycolic acid); Cip, ciprofloxacin; HPAP, 4-(hydroxymethyl) phenylboronic acid pinacol ester; MXF, moxifloxacin; FR, folate receptor; FDKP, N-fumaroylated diketopiperazine; S. pneumoniae, Streptococcu pneumoniae; ICG, indocyanine green; NHS, N-hydroxysuccinimide; PTT, photothermal therapy; MRSA, methicillin-resistant Staphylococcus aureus; K. pneumoniae, Klebsiella pneumoniae; Cpl-1, an endolysin derived from Cp-1 phage; DA, 2,3-dimethyl maleic anhydride; CS, chitoligosaccharide.

### 2.3 Extracellular vesicles

At present, exosomes and microvesicles are the most commonly utilized types of EVs, which are heterogeneous and lipid bilayer-embedded particles secreted actively by cells. EVs vary in size from 30 nm to several micrometers. EVs contain lipids, nucleic acids and proteins. The envelope structure, which affects the stability and targeting of EVs, is made up of enzymes, heat shock proteins (HSPs), membrane proteins (integrins), and lipids (e.g., phosphatidic acid; sphingolipids). EVs are used to deliver miRNAs, mRNAs, etc. to regulate gene expression in target cells. EVs can be chemically modified, have low immunogenicity and high biocompatibility. When combined with various drug delivery methods, EVs can produce effective antimicrobials with positive clinical therapeutic outcomes. They can transport biologically active materials like proteins, lipids, metabolites, and nucleic acids (RNA/DNA). For instance, EVs can transport non-coding RNAs that control gene expression through a selective loading mechanism. This has a significant role in lung infections and injuries and is anticipated to aid in the treatment of bacterial pneumonia (Abreu et al., 2016).

**Figure 4:**
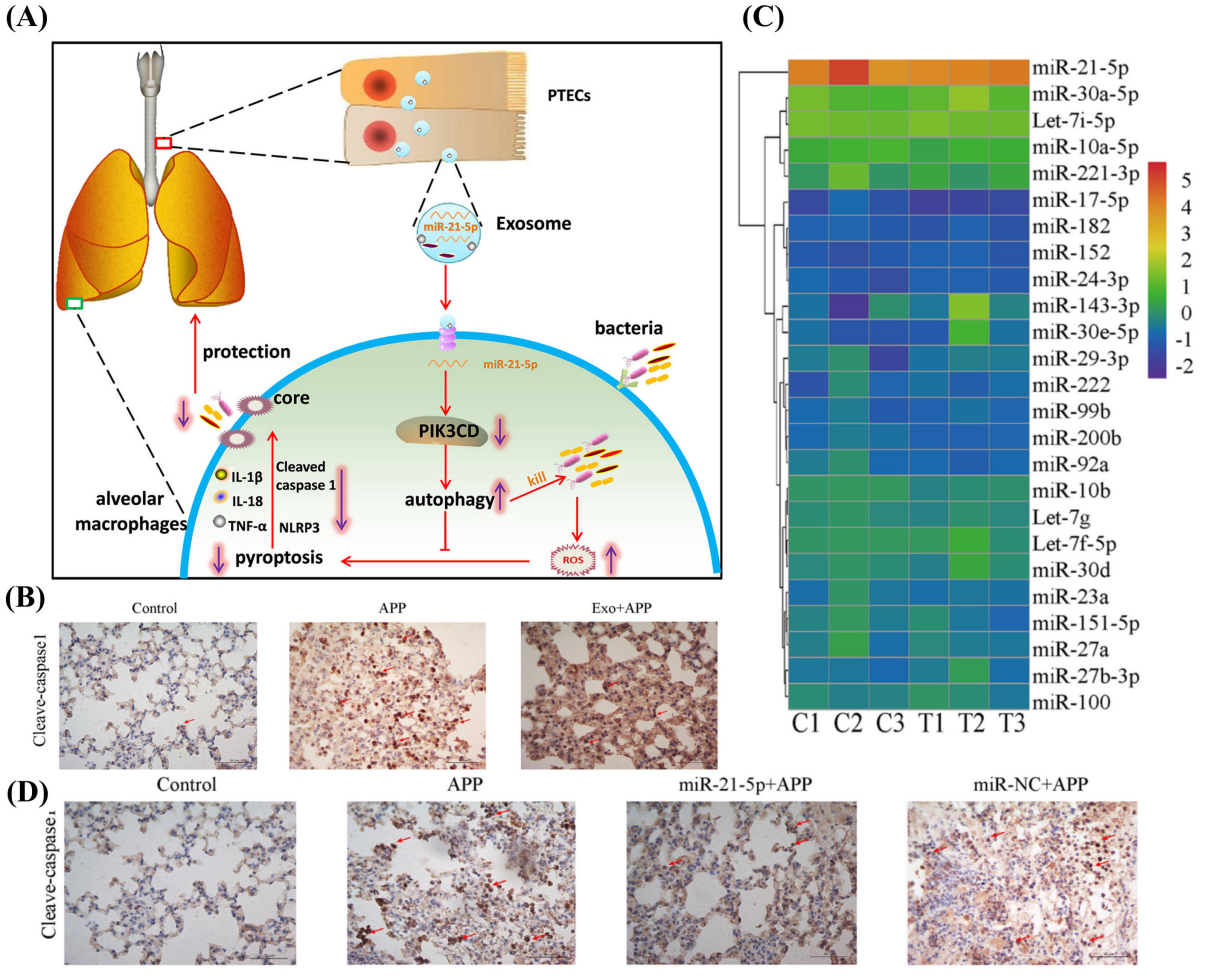
**(A)** Molecular mechanism of PTEC exosomal miR-21-5p inhibits alveolar macrophage pyroptosis to resist pulmonary bacterial infection through PIK3CD-autophagy pathway. Reproduced by permission. Copy right 2024, Elsevier. **(B)** Exosomes administration significantly reduced the extent of pyroptosis. Reproduced by permission. Copy right 2024, Elsevier. **(C)** Among the miRNAs contained in TEC-derived exosomes, miR-21-5p exhibited the highest content. Reproduced by permission. Copy right 2024, Elsevier. **(D)** miR-21-5p significantly reduced the degree of inflammation in lung tissue. Reproduced by permission. Copy right 2024, Elsevier.

Host EVs regulate the immune response through epigenetic modulation to fight bacterial pneumonia. A study by Wang et al. suggests that host-derived EVs contain miRNAs that achieve local immune enhancement by inhibiting macrophage apoptosis(**Figure 4A**). According to a prior study, exosomes derived from tracheal epithelial cells (TECs) have the potential to improve resistance in mice, animal experiments proved it (**Figure 4B**). Deep sequencing indicated that miR-21-5p and/or miR-NC are involved in this phenomenon (**Figure 4C**). TECs were able to secrete exosomes enriched with miR-21-5p that could induce autophagy in alveolar macrophages (AMs) by activating the PI3K/Akt pathway through targeting the PIK3CD gene of the AMs, thereby inhibiting ROS overproduction. Treatment of AMs with synthetic miR-21-5p mimics or miR-NC revealed that miR-21-5p significantly reduced the intracellular bacterial counts of *Actinobacillus pleuropneumoniae* (APP) when compared to miR-NC treatment(**Figure 4D**) through a novel mechanism: the PIK3CD gene in AMs is targeted to activate the PI3K/Akt pathway, which in turn induces autophagy, this inhibits the overproduction of ROS and activates the NLRP3 inflammasome, preventing focal death and improving bacterial clearance. *In vitro* and *in vivo*, this process has been demonstrated to be therapeutic for *MRSA* infections, *Klebsiella pneumoniae*, and APP, offering a novel approach to treat drug-resistant bacterial pneumonia by focusing on the host autophagy-focused death balance (Wang et al., 2024b). Second, variations in EVs composition can serve as biomarkers, and the levels of their surface proteins or miRNAs are strongly linked to the prognosis for sepsis, coagulation abnormalities, or the severity of pneumonia. Third, because of their adjuvant qualities and inherent immunogenicity, outer membrane vesicles are considered as promising vectors for the development of novel vaccines in clinical settings. Bacterial membrane vesicles can be used as a therapeutic agent to generate protective immunity in a vaccine or as a virulence factor carrier to induce the immune response (Behrens et al., 2021). For example, the use of outer membrane vesicle (OMV)-based vaccines which induce protective humoral (IgG/IgA) and cellular (IFN-γ^+^ T cell) responses (Nieves et al., 2011).

Also, therapeutic properties of Mesenchymal Stem Cell-Derived EVs (MSC-EVs) in several lung disorders like acute respiratory distress syndrome, asthma, pulmonary hypertension, and pneumonia, were anti-inflammatory, reparative and immunomodulatory. MSC EVs can deliver small molecule nucleic acid miR-145 that inhibits the expression of multidrug resistance-associated protein 1 (MRP1), that increases production of leukotriene B4 (LTB4) enhancing antimicrobial activity and reducing lung inflammation and infection (Monsel et al., 2015). According to another study, MSC EVs reduced the bacterial load, lung protein permeability, and enhanced alveolar liquid clearance (Park et al., 2019). Since MSC-EVs have the benefits of cell-free therapies, such as preventing immunological rejection and delivering bioactive substances precisely, they offer a novel approach to the precise treatment of pneumonia and have emerged as a highly promising therapeutic approach in regenerative medicine (Abreu et al., 2016; Jung et al., 2021).

## 3 Inorganic nanomaterials

Inorganic nanomaterials, due to their small size, possess a high percentage of surface atoms and a much larger specific surface area, which increases surface energy and improves chemical reactivity. This property makes them particularly amenable to surface modifications, such as coating with polymers or targeting ligands, to improve drug loading capacity. Additionally, their size contributes to greater mechanical strength, lower melting points, and improved stability under physiological conditions.

**Figure 5:**
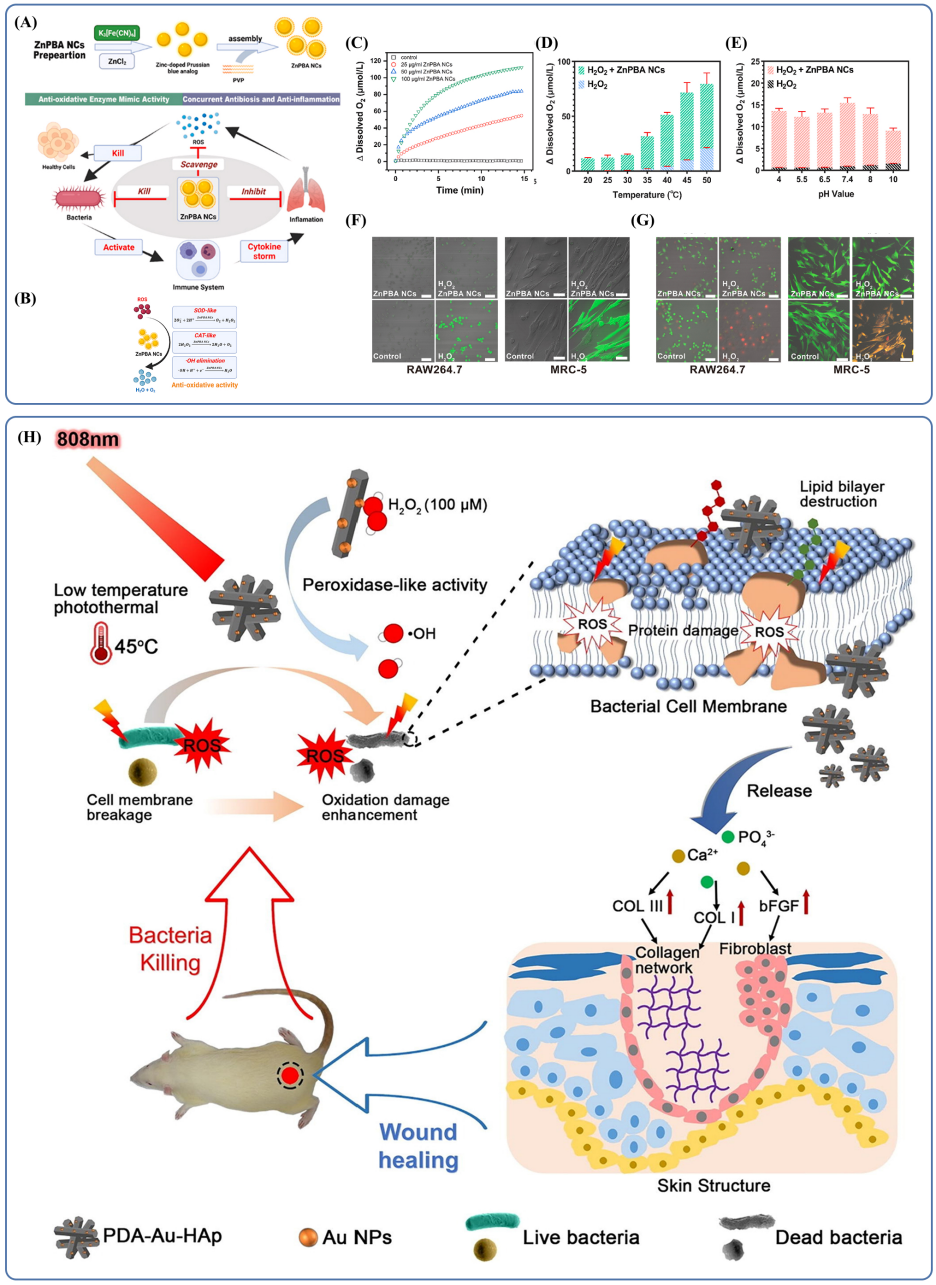
**(A)** Schematic mechanistic illustration of the therapeutics of ZnPBA NCs against acute bacterial pneumonia. Reproduced by permission. Copy right 2022, Elsevier. **(B)** Schematic illustration of antioxidative activities of ZnPBA NCs. Reproduced by permission. Copy right 2022, Elsevier. **(C)** The concentration-dependent catalytic activity of ZnPBA NCs. Reproduced by permission. Copy right 2022, Elsevier. **(D)** The temperature-dependent catalytic activity of ZnPBA NCs. Reproduced by permission. Copy right 2022, Elsevier. **(E)** ZnPBA NCs exhibit high CAT-mimetic activity under different pH value in the range from 4.0 to 10.0. Copy right 2022, Elsevier. **(F)** ZnPBA NCs significantly reduced intracellular oxidative stress levels. Reproduced by permission. Copy right 2022, Elsevier. **(G)** ZnPBA NCs can effectively reduce oxidative stress and prevent cell apoptosis. Reproduced by permission. Copy right 2022, Elsevier. **(H)** Schematic illustration of photothermal therapy combined with catalysis to achieve antibacterial effects and promote wound healing *in vivo*. Reproduced by permission. Copy right 2018, Elsevier.

Their tunable physicochemical properties and stability allow inorganic nanomaterials to perform a variety of roles, including antibacterial, anti-inflammatory, immunomodulatory, and drug delivery functions (Liu et al., 2022; Yang et al., 2020). Inorganic nanomaterials are being explored in several applications. One example is zinc hexacyanoferrate nanocatalysts (ZnPBA NCs), which are self-assembled zinc-doped Prussian blue analogs (Zhang et al., 2021; Liu et al., 2022). These nanocatalysts show consistent scavenging of ROS and direct antibacterial activity (**Figure 5A**). The activity of catalase (CAT) and superoxide dismutase (SOD)-like enzymes is responsible for the effectiveness of these antioxidants, enabling them to neutralize destructive ROS (including H_2_O_2_, superoxide anions), giving them antioxidant effects (**Figure 5B**). The antioxidant capability can endure different temperature, pH levels, and substrate concentrations as shown in (**Figure 5C–E**). ZnPBA NCs have the ability to suppress inflammatory genes along with the oxidation of tissues and cells due to bacterial infections. By lowering the levels of cytokines such as IL-1β, IL-6, and TNF-α and restoring leukocyte counts to normal, they could reduce inflammatory damage (Zhang et al., 2016; Sies and Jones, 2020; Arnold et al., 2001; Jarosz et al., 2017). In cell models exposed to oxidative stress and co-incubated with ZnPBA NCs, fluorescence representing ROS was significantly reduced (**Figure 5F**), and apoptosis was prevented (**Figure 5G**). In a mouse model of acute bacterial pneumonia, ZnPBA NCs effectively reduced disease symptoms. This was evidenced by decreased hemocyte infiltration in H&E-stained lung sections, nearly absent CD45^+^ neutrophils in IHC staining, and lowered levels of inflammatory cytokines TNF-α, IL-1β, and IL-6 in lung tissues (Chu et al., 2015; Ma et al., 2020).

Due to their unique structural features, inorganic nanomaterials have become a widely used carrier platform in many applications. When their size approaches or falls below the wavelength of light, these materials exhibit distinct optical and electrical properties, including photothermal effects, electrical conductivity, and changes in carrier mobility. These characteristics make inorganic nanomaterials a promising foundation for photodynamic therapy (PDT), offering opportunities for combined therapeutic approaches. This concept has already been practiced in dermatology, where such effects not only help eliminate microbes and reduce oxidative stress but also support wound healing by promoting granulation tissue formation and collagen production through gene regulation and other pathways (**Figure 5H**). The same principle holds potential in treating infections at other body sites. For example, researchers have developed drug-loaded gold/silver hybrid nanocages (Au/Ag NCs) (Yang et al., 2020) that possess specialized photodynamic properties. When combined with near-infrared (NIR) radiation, which allows deep tissue penetration, these Au/Ag NCs exhibited effective photothermal conversion. In lung tissue, after HA-P(Au/Ag)/NIR treatment, the localized temperature increased from 33°C to 40.6°C. *In vitro* experiments revealed that treatment with Au/Ag NCs and NIR caused the membranes of multidrug-resistant *Acinetobacter baumannii* (MDR-AB) to shrink with the formation of numerous pores, resulting in severe deformations of the bacteria. Other research has shown that higher temperatures can interfere with metabolic signaling pathways of the bacteria thereby killing the bacteria without damaging the surrounding healthy tissues (Zhang et al., 2019; Pansare et al., 2012; Xu et al., 2019; Korupalli et al., 2017; Qing et al., 2019). The results suggest that photodynamic therapy, which is already used in dermatology, can also be useful in antimicrobial therapy. Inorganic nanomaterials are thus promising materials for various clinics application.

Apart from robust antimicrobial activity, inorganic nanomaterials combine multiple antibacterial mechanisms via surface modifications (Yang et al., 2020). The Au/Ag nanocages were further modified through the conjugation of HA and antimicrobial peptides (APs). HA was attached via electrostatic adsorption and APs through Au-S bonding which assembled a composite with structural and functional dissimilarities (Banerjee et al., 2011; Wan et al., 2013). Au/Ag NCs show good antibacterial effect on MDR-AB by inhibiting and killing at low conc. Experiment results revealed that HA-P(Au/Ag) disrupted the cell membranes of both gram-positive and gram-negative bacteria to inhibit their growth and replication (Martínez-Gutierrez et al., 2012; Lee et al., 2015; Hernandez-Montelongo et al., 2016). The presence of HA increased the binding affinity for MDR-AB, improving targeting capability. In a mouse model of MDR-AB-induced pneumonia, possibly due to poor delivery efficiency, histological analysis revealed that conventional antibiotic treatment failed to achieve effective drug deposition in the lungs. These mice also showed disrupted alveolar structures and signs of erythrocyte leakage. In contrast, the group treated with HA-P(Au/Ag) combined with NIR irradiation displayed preserved alveolar structure and a significant decrease in erythrocyte accumulation. This indicates that HA-P(Au/Ag) not only limits tissue damage but also improves antibacterial performance.

## 4 Organic-inorganic composite nanomaterials

**Figure 6:**
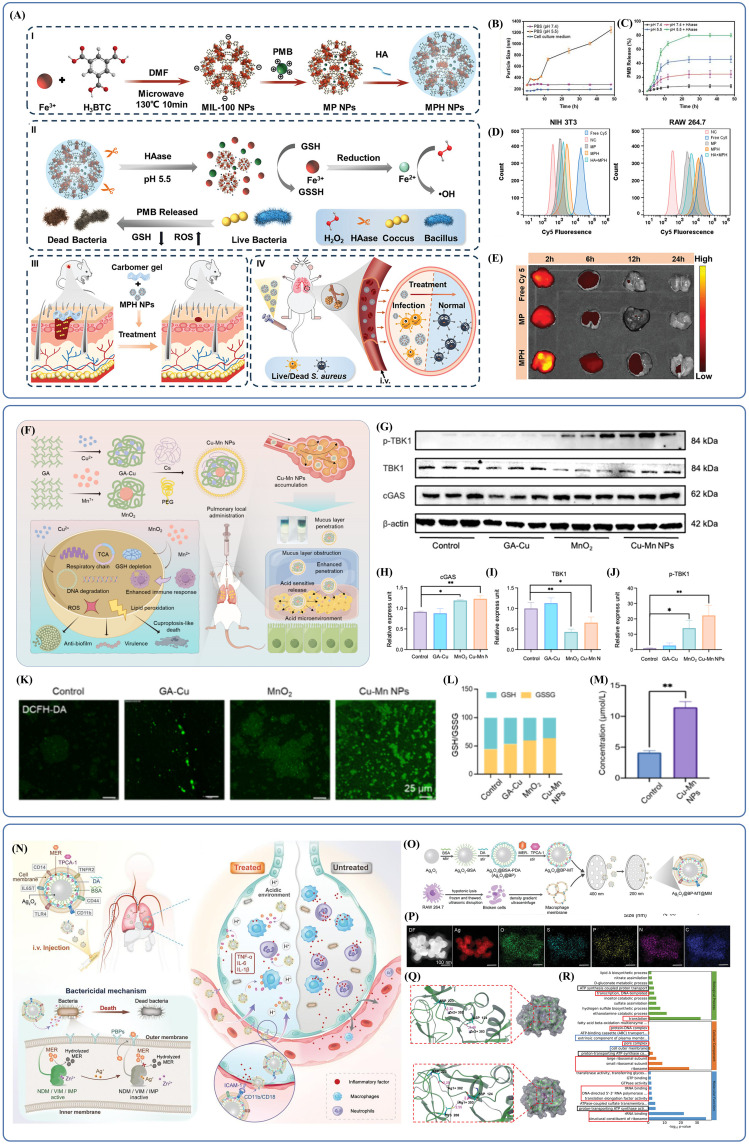
**(A)** Schematic diagram of the synthesis and antibacterial application of MPH NPs. Reproduced by permission. Copy right 2024, Wiley. **(B)** The intercomponent interactions in MPH NPs are weakened and disrupted under acidic conditions, leading to the disintegration of MPH NPs. Reproduced by permission. Copy right 2024, Wiley. **(C)** The presence of acidic conditions and HAase accelerates the release of PMB and enhances the antibacterial effect. Reproduced by permission. Copy right 2024, Wiley. **(D)** MPH NPs specifically bind to and are internalized by CD44-expressing cells. Reproduced by permission. Copy right 2024, Wiley. **(E)** The lungs in the MPH NPs treatment group exhibited strong fluorescence, which lasted for 24 hours. Reproduced by permission. Copy right 2024, Wiley. **(F)** Inhibitory bactericidal activity and therapeutic effect of Cu-Mn NPs mediating acute pneumonia. Reproduced by permission. Copy right 2024, American Chemical Society. **(G-J)** In the MnO_2_ and Cu-Mn NPs groups, cGAS levels were slightly upregulated, while p-TBK1 levels were significantly upregulated. Reproduced by permission. Copy right 2024, American Chemical Society. **(K)** The bacterial membranes in the Cu-Mn NPs group were the most severely damaged. Reproduced by permission. Copy right 2024, American Chemical Society. **(L)** Cu-Mn NPs are capable of disrupting the balance of the GSH/GSSG ratio. Reproduced by permission. Copy right 2024, American Chemical Society. **(M)** The Cu-Mn NPs group showed significant accumulation of Cu. Reproduced by permission. Copy right 2024, American Chemical Society. **(N)** Schematic illustration of biomimetic and responsive nanovesicles Ag_2_O_2_@BP-MT@MM target the infected lung and reverse carbapenem resistance by tackling metallo-β-lactamase (MBL) producing superbugs infections. Reproduced by permission. Copy right 2023, Wiley. **(O)** Schematic illustration of synthetic procedures for Ag_2_O_2_@BP-MT@MM. Reproduced by permission. Copy right 2023, Wiley. **(P)** Silver, oxygen, sulfur, phosphorus, carbon, and nitrogen are uniformly distributed in Ag_2_O_2_@BP-MT@MM. Reproduced by permission. Copy right 2023, Wiley. **(Q)** Different structural bases determine that Ag substitution exhibits higher activity. Reproduced by permission. Copy right 2023, Wiley. **(R)** Ag_2_O_2_@BP-MT@MM exerts a significant effect on the regulation of protein expression. Reproduced by permission. Copy right 2023, Wiley.

The potential of nanotechnology in treating bacterial pneumonia is further enhanced by the combination of organic and inorganic nanomaterials. Centered around an inorganic core, organic-inorganic composite nanomaterials integrate multiple antimicrobial mechanisms from both material types to produce synergistic antimicrobial effects. The use of suitable organic modifications also improves biocompatibility, controlled-release properties and delivery accuracy. Scientists created NPs with merged bacteria-killing mechanisms (MPH NPs) where they loaded PMB on HA-modified iron-based metal-organic frameworks (Fe-MOFs, MIL-100) (Guo et al., 2024). The NPs possess two key antimicrobial mechanisms: chemodynamic therapy and target antibiotic delivery. The first way is to make plenty of hydroxyl radicals (·OH) in the presence of hydrogen peroxide (H_2_O_2_). This damages membrane and intracellular biomolecules. Under acidic conditions, this catalytic efficiency gets even higher to create exact antimicrobial effects at the infection site. Additionally, Fe^3+^ on MIL-100 surfaces can be reduced to Fe^2+^ by glutathione (GSH), consuming local GSH and disrupting the bacterial antioxidant defense system. The latter mechanism is triggered by the acidic microenvironment (pH =5.5), which promotes MIL-100 framework degradation, while hyaluronidase (HAase) simultaneously degrades the surface HA layer. This dual-triggered degradation accelerates PMB release, achieving high localized antibiotic concentrations. HA modification significantly improves nanoparticle stability and biocompatibility, enabling effective *in vivo* therapeutic action. Collectively, this synergistic approach depletes GSH, generates substantial ROS, and enhances PMB’s bactericidal efficacy through membrane disruption (**Figure 6A**). Regarding material design, incubation studies in phosphate-buffered saline (PBS) demonstrated that HA coating substantially enhanced MPH NPs stability (**Figure 6B**), while acidic conditions and elevated HAase concentrations accelerated PMB release (**Figure 6C**), confirming superior targeting performance. Distribution and uptake studies revealed distinct internalization of Cy5-labelled MPH NPs by LPS-stimulated RAW 264.7 cells (CD44-expressing) (Vachon et al., 2006) compared to NIH 3T3 (non-CD44-expressing) (Rios de la Rosa et al., 2017) cells, suggesting specific binding affinity of MPH NPs to CD44-positive cells (**Figure 6D**). Dynamic fluorescence intensity analysis indicated sustained strong pulmonary fluorescence in the treatment group for 24 hours following administration of labelled MPH NPs (**Figure 6E**), highlighting prolonged action and cumulative therapeutic effects, thus underscoring its promise as an effective therapeutic strategy.

It is crucial to highlight that inorganic nanomaterials, especially those relying excessively on ROS-based mechanisms, may have limited effectiveness against aerobic bacteria highly resistant to ROS (Ashley et al., 2020; Dickson et al., 2016; Gaupp et al., 2012). Therapeutic approaches that depend heavily on ROS-induced antimicrobial activity risk the early emergence of resistance due to reliance on a single bactericidal mechanism, restricting their application potential. An innovative solution involves strategically selecting central metal ions to diversify antibacterial mechanisms. Hua et al. developed copper-based composite antibacterial nanoparticles (Cu-Mn NPs) by synthesizing a copper–gallic acid (GA-Cu) complex through metal–polyphenol network formation, incorporating manganese dioxide (MnO_2_), and modifying nanoparticle surfaces with chitosan (Cs) and PEG to produce positively charged, hydrophilic organic-inorganic composite NPs (Hua et al., 2024)(**Figure 6F**). Unlike traditional inorganic materials that rely predominantly on ROS generation, Cu-Mn NPs depend on Mn ions released in local tissues to enhance the antimicrobial immune response through stimulation of interferon (IFN) and pro-inflammatory cytokines via the cGAS-STING signaling pathway. This mechanism was corroborated by WB analyses, which revealed increased expression levels of cGAS-STING pathway-associated proteins in the MnO_2_ and Cu-Mn NPs treatment groups (**Figure 6G-J**). Additionally, MnO_2_ functions as a GSH-depleting agent, as evidenced by the strongest green fluorescence (representing ROS) and significant disruption of the GSH/GSSG ratio observed in the Cu-Mn NPs group (**Figure 6K-L**). As a central component, copper ions were stably loaded onto the NPs and rapidly released in acidic infections microenvironments, inducing copper-dependent bacterial cell death. Recent studies indicate that copper induces bacterial death upon accumulation by interacting with components of the tricarboxylic acid cycle (TCA) and the respiratory chain, particularly affecting cells reliant on aerobic respiration (Tsvetkov et al., 2022; Huang et al., 2024; Zhou et al., 2023; Lu et al., 2024; Mei et al., 2023). Cu accumulation was also observed in bacteria treated with Cu-Mn NPs compared to controls (**Figure 6M**). Although the inorganic component exhibited a unique bactericidal mechanism, drug delivery was initially impeded by the pulmonary mucus barrier. Surface modification with positively charged chitosan enhanced NPs binding affinity to negatively charged bacterial membranes, while PEG coating improved hydrophilicity, facilitating mucus penetration and enhancing targeted delivery, thus reducing potential copper toxicity. Consequently, Cu-Mn NPs represent a promising composite material with high therapeutic potential.

Another intriguing study focused on designing materials to overcome bacterial resistance (Li et al., 2024a)(**Figure 6N**). Researchers constructed biomimetic, responsive silver peroxide-based nanovesicles (Ag_2_O_2_@BP-MT@MM), employing silver peroxide (Ag_2_O_2_) NPs as the core structure. The NPs were surface-modified with bovine serum albumin (BSA) and polydopamine (PDA) to encapsulate the antibiotic meropenem (MER) and the anti-inflammatory drug TPCA-1, forming Ag_2_O_2_ @BP-MT. Subsequently, macrophage membranes (MM) were incorporated to enable targeted delivery to inflamed lung tissues (**Figure 6O**). Elemental mapping confirmed the homogeneous distribution of Ag, oxygen, sulfur, phosphorus, carbon, and nitrogen within Ag_2_O_2_ @BP-MT@MM (**Figure 6P**). The principal mechanism of action involved acid-responsive decomposition of Ag_2_O_2_ NPs, releasing Ag^+^ ions, which effectively displaced Zn^2+^ from the active sites of metallo-β-lactamases (MBLs), including NDM-1, VIM, and IMP. This displacement inhibited enzyme activity, restoring bacterial susceptibility to meropenem. Structural analyses elucidated the underlying mechanism: Zn^2+^ typically occupied the active pocket of NDM-1, forming salt bridges with residues ASP223 and ASP124, whereas Ag^+^ interacted more robustly by establishing salt bridges with ASP223, ASP124, and CYS208 residues. Further investigations demonstrated that Ag^+^ exhibited superior inhibitory capacity against MBL activity relative to Zn^2+^, supported by the lower binding free energy and more stable complex formation with NDM-1 (**Figure 6Q**). Both *in vitro* and *in vivo* experiments revealed that Ag^+^ synergized with meropenem to eradicate bacteria and delay resistance emergence by suppressing resistance gene expression. The biomimetic MM endowed the material with targeted delivery capabilities through specific interactions between membrane-bound CD11b/CD18 and ICAM-1 on inflamed endothelial cells. Additionally, the membrane adsorbed LPS and pro-inflammatory cytokines, providing dual inflammatory control. The encapsulated TPCA-1 further suppressed inflammation by inhibiting the NF-κB signaling pathway, attenuating excessive inflammatory responses. RNA-seq analysis indicated that Ag_2_O_2_ @BP-MT@MM exerted antimicrobial effects by regulating protein expression (including reducing drug-resistance enzyme expression), altering energy metabolism, and influencing the synthesis of structurally related proteins (**Figure 6R**). Ultimately, the modification with biological proteins and macrophage membranes imparted exceptional biocompatibility, highlighting the substantial potential of Ag_2_O_2_ @BP-MT@MM in preventing drug resistance emergence and combating drug-resistant bacteria.

## 5 Discussion

Nanotechnology has shown considerable promise in the treatment of bacterial pneumonia. By encapsulating drugs or active agents within NPs, their release can be controlled, preventing premature loss and increasing therapeutic effectiveness at the target site due to the structural advantages of the nanocarrier. However, several key issues remain. One major concern is the limited spatiotemporal specificity of current nanomaterial systems. Although various mechanisms have been designed to improve targeting precision, the control is not yet sufficient. As noted earlier, some nanomaterials help reduce tissue injury by suppressing ROS and dampening immune responses, while others rely on ROS generation and immune activation to exert antibacterial effects. This apparent contradiction highlights the need for finely tuned timing and localization. During the early phase of infection, an increase in immune activity and ROS can help eliminate pathogens, but once bacterial loads are reduced or cleared, these same responses must be downregulated to avoid further tissue damage. Suppressing ROS at later stages promotes tissue repair and recovery. Therefore, nanomaterials must not only reach the target site but also adjust their function based on the stage of infection. To achieve this goal, methods such as precise dosage control, the use of responsive carriers, and the ability to dynamically interact with the changing microenvironment at the infection site can be applied. Another example is the use of organic-inorganic composite nanomaterials such as Cu-Mn NPs, which can induce copper-mediated cell death by disrupting aerobic respiration in bacteria. However, these materials must be designed with care to avoid damaging normal lung tissue. Such considerations are critical in the design of safe and effective nanomaterials.

The comparison between liposomes and EVs has also attracted researchers’ interest. Similar to liposomes, EVs are also designed based on biological membranes. EVs induce biological responses through intercellular signaling, such as miRNA, mRNA, etc., achieving regulation of target cells or tissues. This means they do not require artificial loading, preserving biological activity more completely, especially in the delivery of nucleic acid-based drugs, where they have irreplaceable advantages. Besides, since exosomes are derived from endogenous cellular secretion, their membrane structure is highly homologous to the host cell, preventing rapid metabolic clearance and resulting in lower toxicity (Bai et al., 2025). In terms of targeting, extracellular vesicles have corresponding ligands on their surface, and through surface binding and endocytosis, they naturally have a delivery advantage (Han et al., 2021b; Zheng et al., 2025). In contrast, liposomes preferentially follow the distribution in the circulatory system mainly in organs like the liver and spleen, leading to targeting issues. Targeting can only be achieved through modifications and changes in components, which raises concerns related to immunity. However, this does not mean that EVs are perfect delivery carriers, as challenges such as large-scale production, standardization of separation techniques, and batch-to-batch reproducibility remain key obstacles for clinical translation (Wang et al., 2021).

Safety remains a central concern in the development and clinical application of nanomaterials. While the overall safety profiles of these materials vary, some, like liposomes and polymeric NPs, have been studied extensively and are approved in specific clinical contexts. Still, limitations exist. Liposomal systems often have limited drug loading capacity, also may lead to leakage or abnormal release rate, affecting treatment outcomes (Bozzuto and Molinari, 2015). Polymeric nanomaterials, particularly those modified with HA, may face challenges in aligning degradation rates with the patient’s disease progression, resulting in mismatched drug release timing (Engström-Laurent, 1997). Modifications involving host-derived components or EVs may also introduce immune-related risks. Inorganic nanomaterials pose additional challenges. Their potential for size-dependent toxicity, mainly mechanism mediated by oxidative stress, raises safety concerns (Carlson et al., 2008). Furthermore, poor degradability can result in long-term *in vivo* accumulation, which may increase the risk of pulmonary fibrosis, chronic toxicity, or other adverse outcomes.

The heterogeneity of bacterial pneumonia presents another major challenge. Differences in pathogens, infection sites, and the immune status of individual patients demand that nanomaterials be highly adaptable and capable of modification. Some organic nanomaterials, although generally low in toxicity, lack the mechanical strength to withstand the *in vivo* environment and show poor drug-loading capacity due to their structural and physicochemical limitations (Azadi et al., 2024). The high cost of designing, developing, and producing customized nanocarriers further limits their broader clinical adoption. In addition, large-scale manufacturing techniques remain underdeveloped. Common pulmonary drug delivery methods, such as nebulization, still face issues with uniformity and deep tissue penetration (Yu et al., 2023).

However, one advantage is that nanomaterial design does not always need to start from zero. With appropriate adaptations and by designing on existing technologies, new and unexpected outcomes can be achieved. For example, liposomal systems originally designed for liver targeting can be modified to redirect activity toward the lungs, often with improved therapeutic outcomes. Similarly, nanomaterials developed for dermatology, particularly those involving photodynamic therapy, can be repurposed for pulmonary treatment. Inorganic NPs with strong photothermal effects, already effective in skin applications, can be used to build lung-targeted photodynamic platforms when combined with deep-penetrating laser irradiation.

From a methodological standpoint, verifying safety is essential before any material is applied in humans. All new treatment strategies must undergo rigorous evaluation, including preclinical testing, multi-phase and multi-center randomized controlled clinical trials (RCTs), and post-market monitoring. High-quality, evidence-based approaches are required, not just reliance on animal models, to confirm a material’s effectiveness and safety.

For instance, the long-term safety and biocompatibility of inorganic nanomaterials have yet to be systematically assessed. Existing short-term toxicity studies, often lasting only days or weeks, do not provide sufficient evidence to support clinical translation. Although a large number of preclinical studies have achieved ideal results in material design, mechanism selection, delivery, and therapeutic effects, the inability to translate these results into clinical applications is one of the reasons why nanomaterials are rarely used in the treatment of bacterial pneumonia. Moreover, the nanomaterials used in clinical applications have relatively simple structures, suggesting that some structural modifications that show superior effects in preclinical studies do not necessarily guarantee stable and effective clinical outcomes. This may be due to the insufficient sample size in preclinical studies, physiological and pathological differences between humans and experimental animals, and methodological shortcomings that were either not reported or not tracked over the long term in earlier studies. Among all the materials mentioned in this paper, liposomes are a relatively mature application. In the first-in-human, double-blind, placebo-controlled, randomized trial conducted in ICU wards in France and Belgium, liposomes, known as CAL02, had a surface with numerous microdomains designed to capture toxins (Laterre et al., 2019). These liposomes were used to capture cytotoxins, downregulate inflammation, and could be used in the treatment of bacterial pneumonia. The patients enrolled were ICU patients diagnosed with community-acquired pneumonia caused by *Streptococcus pneumoniae*. The first phase randomly assigned 6 patients (1:1) to either the low-dose CAL02 group or the placebo group, while the second phase randomly assigned 18 patients (14:4) to either the high-dose CAL02 group or the placebo group. From baseline to day 8, the combined CAL02 group saw an average reduction of 60.2% in the APACHE II score, while the placebo group had a reduction of 22.1% (15.5-28.7). The combined CAL02 group saw an average reduction of 65.0% (50.7-79.4) in the SOFA score, while the placebo group had a reduction of 29.2%. Besides the scale scores, the combined treatment group showed significant improvements in imaging, serology, and hospitalization-related indicators. The adverse effects detected during this process were mainly elevated transaminases, but they were found to be related to the patient’s underlying conditions, indicating good drug tolerance. A limitation of the study was the small sample size and the lack of investigation into whether CAL02’s efficacy is concentration-dependent. Overall, the drug still shows significant potential in managing systemic inflammation in bacterial pneumonia patients and provides confidence for the clinical translation of nanomaterials.

Last but not least, the regulations, laws, and moral limitations pertaining to nanotechnology are still developing. Since nanotechnology still needs systematic clinical trials before clinical use and cannot be substituted for traditional animal experiments, more ethical and regulatory discussions and norms are required before ensuring their safe, effective, and legal application in the treatment.

## 6 Conclusion

This review provides an overview of the development of nanotechnology in the management of bacterial pneumonia, highlighting its notable benefits in enhancing therapeutic effectiveness, combating drug resistance, and minimizing adverse effects. Nanotechnology significantly supports bacterial pneumonia treatment through targeted drug delivery, improved drug stability, immune response modulation, and inherent antibacterial and anti-inflammatory properties. Furthermore, the combination of organic and inorganic nanomaterials increases the potential of nanotechnology and offers solutions for problems with material toxicity and drug resistance. Numerous challenges still need to be overcome before nanotechnology can be used in clinical practice, such as ensuring the biosafety of nanomaterials, optimizing drug loading and release mechanisms, and facilitating large-scale production. It is predictable that nanotechnology exhibits a wide range of potential applications in the management of bacterial pneumonia and is anticipated to be employed as a safer and more effective therapeutic option in clinical practice.
